# Prognostic factors for overall survival in patients with metastatic castration-resistant prostate cancer treated with radium-223: a meta-analysis of real-world evidence

**DOI:** 10.3389/fonc.2025.1672802

**Published:** 2025-11-19

**Authors:** Baolin Song, Huan Shao, Yanmei He, Xinwei Zhu, Pengfei Qin

**Affiliations:** Department of Urology, Jiaxing Hospital of Traditional Chinese Medicine, Jiaxing, China

**Keywords:** prognostic factor, overall survival, radium-223, castration-resistant prostate cancer, real-world

## Abstract

**Background:**

Metastatic castration-resistant prostate cancer (mCRPC) remains a lethal disease with limited treatment options. Radium-223 (Ra-223) improves survival in bone-predominant mCRPC, but real-world outcomes vary widely. This meta-analysis synthesizes real-world evidence to identify prognostic factors for overall survival (OS) in Ra-223-treated patients.

**Methods:**

Following PRISMA guidelines, we systematically searched PubMed, Embase, Web of Science, and Cochrane Library for observational studies reporting OS-associated prognostic factors in mCRPC patients receiving Ra-223. Pooled hazard ratios (HRs) were calculated. Study quality was assessed via Newcastle-Ottawa Scale.

**Results:**

Among 25 studies (n=8,795 patients), the pooled Ra-223 completion rate was 52.6% (95% CI: 48.9–56.3%). Each additional Ra-223 injection significantly improved OS (HR = 0.478, 95% CI: 0.362–0.630). Poorer OS correlated with older age (HR = 1.012/year), higher ECOG (HR = 2.078), elevated baseline PSA (HR = 1.922), ALP (HR = 1.981), LDH (HR = 1.702), NLR (HR = 2.255), and visceral metastases (HR = 2.342). Protective factors included hemoglobin levels (HR = 0.756/g/dL) and PSA/ALP declines during therapy (HR = 0.386 and 0.701, respectively). Prior chemotherapy predicted worse outcomes (HR = 1.425), while Gleason score and concurrent bone protectants showed no significant association.

**Conclusion:**

Real-world data confirm Ra-223’s survival benefit is closely associated with treatment completion and baseline clinical factors. The findings support risk-stratified patient selection and tailored management in mCRPC.

## 1 Introduction

Prostate cancer remains a global health challenge, ranking as the second most common malignancy in men worldwide (1). While many cases are diagnosed at localized stages, approximately 8% of patients present with metastatic disease at initial diagnosis (2). Furthermore, a significant proportion of men treated for early-stage prostate cancer—estimated at 10-20% within five years of primary therapy—progress to castration-resistant prostate cancer (CRPC), with metastatic CRPC (mCRPC) representing an advanced disease state associated with particularly poor outcomes and limited survival (3, 4). This clinical trajectory underscores the critical need for effective therapeutic strategies to improve outcomes in this challenging patient population.

The development of Ra-223 marked a significant advancement in mCRPC treatment based on the landmark ALSYMPCA trial findings (5). As a targeted alpha therapy, Ra-223 uniquely addresses the complex needs of patients with bone-predominant mCRPC by selectively delivering radiation to osteoblastic metastases while sparing healthy tissues. The ALSYMPCA trial demonstrated not only improved overall survival but also meaningful delays in skeletal-related events, establishing Ra-223 as an important therapeutic option. However, real-world clinical experience has revealed considerable variability in treatment responses, with some patients deriving substantial benefit while others show limited therapeutic response (6–10). This heterogeneity highlights the pressing need to identify reliable prognostic factors that can guide treatment selection and optimize outcomes in clinical practice.

Current understanding of prognostic factors for Ra-223 therapy remains fragmented across studies of varying quality and sample sizes. While some investigations have identified potential predictors such as baseline alkaline phosphatase levels, treatment completion rates, or hemoglobin concentrations, the evidence lacks systematic synthesis and often fails to account for potential confounding variables. Moreover, there is limited consensus on the relative importance of different prognostic markers. This meta-analysis therefore aims to quantify and compare the prognostic impact of specific clinical and biochemical variables on overall survival in mCRPC patients receiving Ra-223 therapy. Prior meta-analyses on prognostic factors in prostate cancer have primarily focused on other treatment modalities, such as androgen receptor pathway inhibitors and Lu-PSMA radioligand therapy (11, 12). This study complements the existing evidence by providing a focused synthesis of real-world evidence for Ra-223.

## 2 Methods

This meta-analysis followed the Preferred Reporting Items for Systematic Reviews and Meta-Analyses (PRISMA) guidelines (13) and was designed to evaluate prognostic factors associated with overall survival (OS) in patients with mCRPC treated with Ra-223 in real-world settings. The study adhered to the following PICOS framework:

Population (P): Patients diagnosed with mCRPC and bone metastases.

Intervention (I): Treatment with Ra-223.

Comparators (C): Not applicable, since single-arm cohort study can provide sufficient evaluation for prognostic factors.

Outcomes (O): Primary outcome was OS, measured by hazard ratios (HRs) for prognostic factors. Secondary outcomes included Ra-223 treatment completion rates.

Study design (S): Real-world observational studies (retrospective or prospective cohorts).

### 2.1 Database searching

A comprehensive systematic literature search was conducted in PubMed/MEDLINE, Embase, Web of Science, and the Cochrane Library from their inception through May 31, 2025, restricted to English-language publications. Grey literature, such as conference abstracts, was not included in this meta-analysis. The search strategy employed a combination of controlled vocabulary terms and free-text keywords including: “radium”, “radium 223”, “radium-223”, “Ra-223” or “Ra 223” for the intervention; these were combined with terms for the target population (“prostate” or “CRPC”). The detailed exact Boolean strings for literature search in these databases are provided in **Supplementary Table S1**. In addition to database searches, we manually examined reference lists of all included studies to identify potentially eligible publications that might have been missed by the electronic searches.

### 2.2 Eligibility criteria

Studies were included if they met the following criteria: (1) enrolled patients with mCRPC and bone metastases who received Ra-223 therapy; (2) reported the HRs and 95% confidence interval for prognostic factors associated with OS; (3) real-world observational studies (retrospective or prospective cohorts); and (4) were published in English. Exclusion criteria: (1) duplicate records; (2) non-English publications; (3) studies with fewer than 100 patients were excluded to ensure robust sample sizes; (4) meta-analyses, reviews, case reports, conference abstracts, letters and animal studies.

### 2.3 Study selection

All identified records from database searches were imported into EndNote X9, where duplicate publications were automatically removed followed by manual verification. The study selection process was conducted in two phases using the PRISMA framework. Initially, two independent reviewers screened all retrieved records by title and abstract to identify potentially eligible studies. In cases of disagreement, a third reviewer was consulted to reach consensus. Subsequently, full-text articles were thoroughly evaluated against the predefined eligibility criteria.

### 2.4 Outcomes and data collection

The primary outcome of this meta-analysis was prognostic factor for OS. Secondary outcome was Ra-223 treatment completion rates (proportion of patients receiving all six planned injections). For prognostic factor analysis, we extracted unadjusted HRs when available, with priority given to estimates for real-world practice. Two independent investigators extracted data using a standardized EXCEL form. The collected data included study characteristics, patient demographics, treatment details, with particular focus on hazard ratios for prognostic factors. All extracted data underwent cross-verification and quality checks, with discrepancies resolved through consensus discussion. For dichotomous outcome, the number of events and the total sample size were recorded. For time-to-event outcome, hazard ratios (HRs) and their corresponding 95% confidence intervals (CIs) were extracted.

For continuous variables (e.g., hemoglobin levels), while some studies reported HRs per unit increase (e.g., +1 g/dL), others dichotomized these variables into higher *vs*. lower levels using study-specific thresholds. For this instance, we extracted all reported HRs (higher *vs*. lower) regardless of the original cutoff values used in individual studies, and performed meta-analyses by treating these as generic comparisons of higher versus lower categories.

For dichotomous variables, some studies reported HRs for “higher *vs*. lower” groups, while others might report “lower *vs*. higher” comparisons. If a study reported HR for the “lower *vs*. higher” group, we took the reciprocal (i.e., 1/HR) to convert it to a “higher *vs*. lower” HR to ensure consistency in meta-analysis. The corresponding 95% CIs were similarly transformed by inverting the original upper and lower limits.

### 2.5 Risk of bias assessment

The methodological quality of included studies was assessed using the Newcastle-Ottawa Scale (NOS) for cohort studies (14), which evaluates three key domains: selection of study groups, comparability of groups, and ascertainment of outcomes. Two reviewers independently scored each study, with discrepancies resolved through discussion or consultation with a third reviewer. The NOS assigns a maximum of 9 stars, with studies receiving ≥7 stars considered high quality, 5–6 stars moderate quality, and ≤4 stars low quality. Since the included studies lacked control groups due to the design of this meta-analysis, the maximum possible score was 7 (excluding the 2 stars normally allocated for comparability between groups). Regarding publication bias assessments, the Egger’s test would be performed when ≥10 studies were available for the primary outcomes, and funnel plots would be plotted and assessed for asymmetry.

### 2.6 Statistical analysis

The statistical analysis was performed using R (version 4.3.3). For the meta-analysis of Ra-223 completion rates, we utilized the metaprop function in R with Freeman-Tukey double arcsine transformation (sm=“PFT”). For the meta-analysis of HRs for overall survival, all HRs were log-transformed prior to analysis to approximate normal distributions, with results subsequently back-transformed to the original scale for clinical interpretation. Fixed-effects models were employed when the I² value indicated low to moderate heterogeneity (<50%), while random-effects models were applied when substantial heterogeneity was present (I² ≥50%). Sensitivity analyses, subgroup analyses, cumulative meta-analyses (conducted using the metacum function), and publication bias assessments would be performed when ≥10 studies were available for a given analysis.

## 3 Results

### 3.1 Characteristics of included studies and study quality

**Figure 1:**
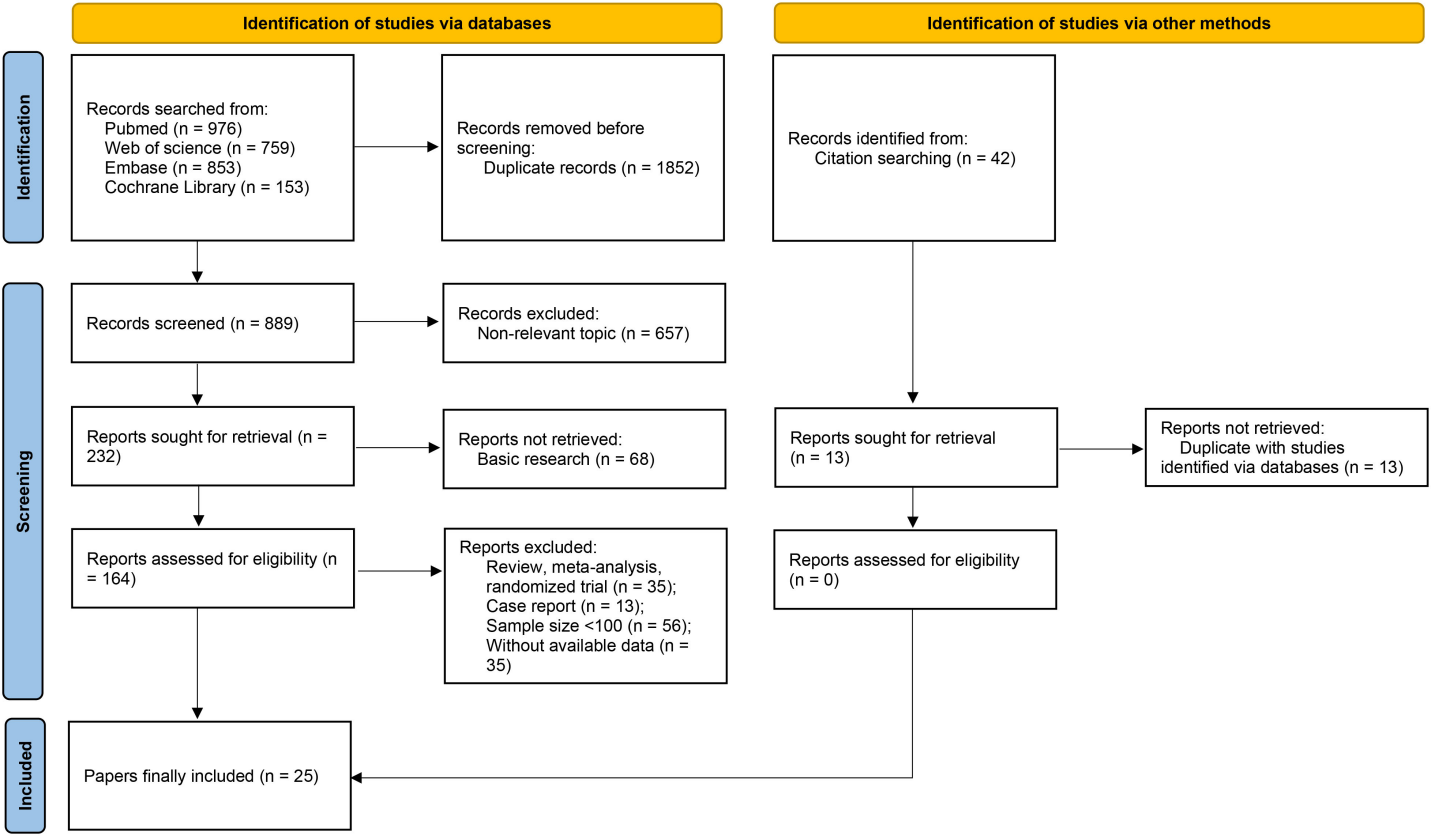
Flow diagram of study selection for the meta-analysis.

As depicted in **Figure 1**, a total of 2,741 records were identified through database searches. After removing duplicates and screening titles and abstracts, 164 full-text articles were assessed for eligibility. Following full-text review, 25 studies were included in the final meta-analysis based on predefined inclusion and exclusion criteria (6–10, 15–34).

The characteristics of the included studies are summarized in **Table 1**. The studies were published between 2015 and 2025 and comprised a cumulative population of 8,795 patients with mCRPC treated with Ra-223. Sample sizes ranged from 100 to 1,376 participants. The majority of studies were retrospective (n = 21), and most were conducted in a multicenter setting (n = 18). The studies were geographically diverse, with contributions from the USA, Italy, Canada, Spain, the UK, the Netherlands, Brazil, and Sweden. Mean or median age across study populations ranged between 67 and 75 years. Reported Ra-223 treatment completion rates varied, with some studies not providing this information (marked as “NR”).

**Table 1:** Characteristics of included studies.

Author	Number of patients	Year	Country	Setting	Design	Age	Ra-223 completion rate	NOS
Etchebehere et al. (6)	110	2015	USA	S	R	69	52.7%	6
Alva et al. (7)	145	2017	USA	M	R	72	51.0%	6
Parikh et al. (8)	189	2018	UK	M	R	72	NR	7
Zhao et al. (9)	318	2020	USA	M	R	67	NR	6
Cheng et al. (10)	198	2019	Canada	M	R	75	46.5%	7
Frantellizzi et al. (15)	NA	2019	Italy	M	R	73.8	NA	6
Badrising et al. (16)	300	2020	Netherlands	M	P	73.6	46.3%	7
Frantellizzi et al. (17)	NA	2021	Italy	M	R	74.1	NA	6
Jiang et al. (18)	228	2020	UK	S	P	72	NR	6
Kuppen et al. (19)	285	2020	Netherlands	M	R	NR	47.4%	7
Frantellizzi et al. (20)	430	2020	Italy	M	R	74.1	61.6%	7
Doelen et al. (21)	180	2021	Sweden	M	R	71	61.1%	6
Al-Ezzin et al. (22)	150	2021	Canada	S	R	74	NR	6
Bauckneht et al. (23)	519	2022	Italy	M	R	74	NR	7
Charrois-Durand et al. (24)	133	2022	Canada	S	R	72	57.9%	6
George et al. (25)	1180	2022	USA	M	R	73	46.0%	6
Kaulanjan et al. (26)	319	2022	Canada	S	R	72	NR	7
Feo et al. (27)	NA	2023	Italy	S	R	73.6	NA	6
Romero-Laorden et al. (28)	169	2024	Spain	S	P	74.4	48.5%	7
Anido-Herranz et al. (29)	145	2024	Spain	M	R	74	57.2%	7
Cruz-Montijano et al. (30)	100	2024	Spain	M	P	72.7	44.0%	7
Feo et al. (31)	581	2025	Italy	M	R	72	63.7%	6
Raval et al. (32)	1376	2025	USA	M	R	68	NR	6
Souza et al. (33)	308	2025	Brazil	M	R	74.6	50.6%	7
Zhou et al. (34)	1062	2025	USA	M	R	75	NR	6

R: retrospective; P: prospective; S: single center; M: multicenter; NR: not reported; NA: not applicable; NOS, Newcastle-Ottawa Scale.

NA indicates that the study was excluded from pooled analysis for certain variables (e.g., patient number or Ra-223 completion rate) to avoid duplication, as it originated from the same research team that had published a more updated dataset.

NR indicates that the variable was relevant but not explicitly reported in the study.

Special consideration was given to multiple publications from the same research groups. Specifically, three studies were authored by the Frantellizzi et al. group (15, 17, 20) and two by the Feo et al. group (27, 31). To minimize patient overlap and data duplication, only the most recent publication from each group was used for extracting data on total patient count, Ra-223 completion rate, and the effect of prognostic factors on OS. Earlier publications from these groups were included only if they reported prognostic factors not covered in the more recent articles.

Study quality was assessed using the NOS, and all studies received a score of 6 or 7. Considering that included studies were single-arm cohorts (for which the maximum NOS score is 7), all were deemed to be of high methodological quality.

### 3.2 Completion of Ra-223

**Figure 2:**
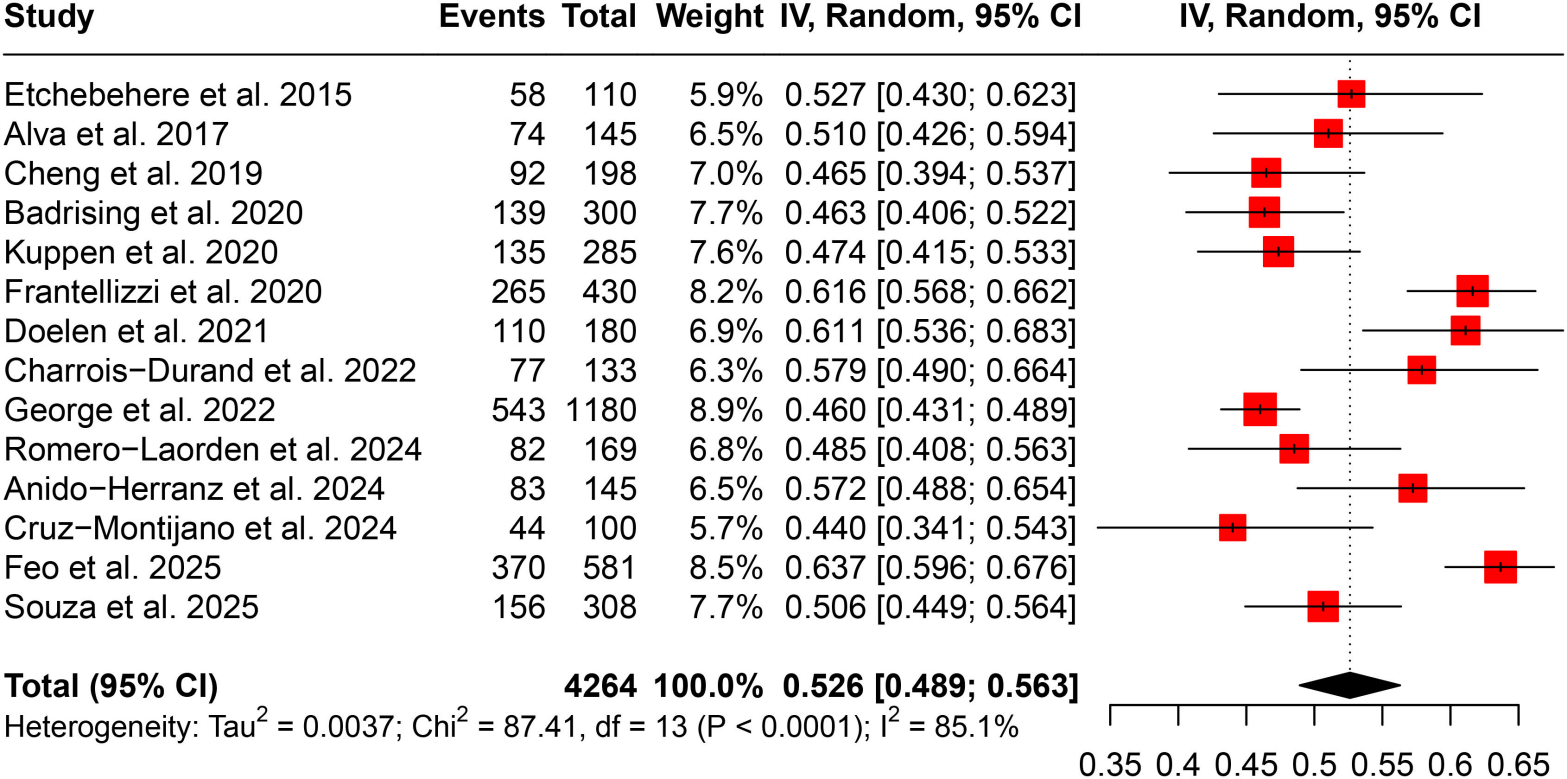
Forest plot of Ra-223 treatment completion rates across included studies.

Completion of Ra-223 treatment was defined as receiving all six planned injections. As shown in **Figure 2**, the pooled completion rate across all included studies was 52.6% (95% CI: 48.9%–56.3%). However, there was significant heterogeneity among studies (I² = 85.1%), indicating substantial variability in completion rates across different cohorts.

Sensitivity analysis demonstrated the robustness of the pooled estimate, with no single study exerting a disproportionate influence on the overall result (**Supplementary Figure S1A**). Subgroup analysis by country (**Supplementary Figure S1B**) revealed that the highest completion rates were reported in Italy (62.8%) and Sweden (61.1%), while the lowest was observed in the United States (48.3%). Cumulative meta-analysis over time (**Supplementary Figure S1C**) did not show a consistent trend of increasing or decreasing completion rates with the year of publication. Assessment of publication bias using a funnel plot (**Supplementary Figure S1D**) did not indicate significant asymmetry, and Egger’s test confirmed the absence of substantial publication bias (P = 0.734).

### 3.3 Prognostic factors associated with OS

**Figure 3:**
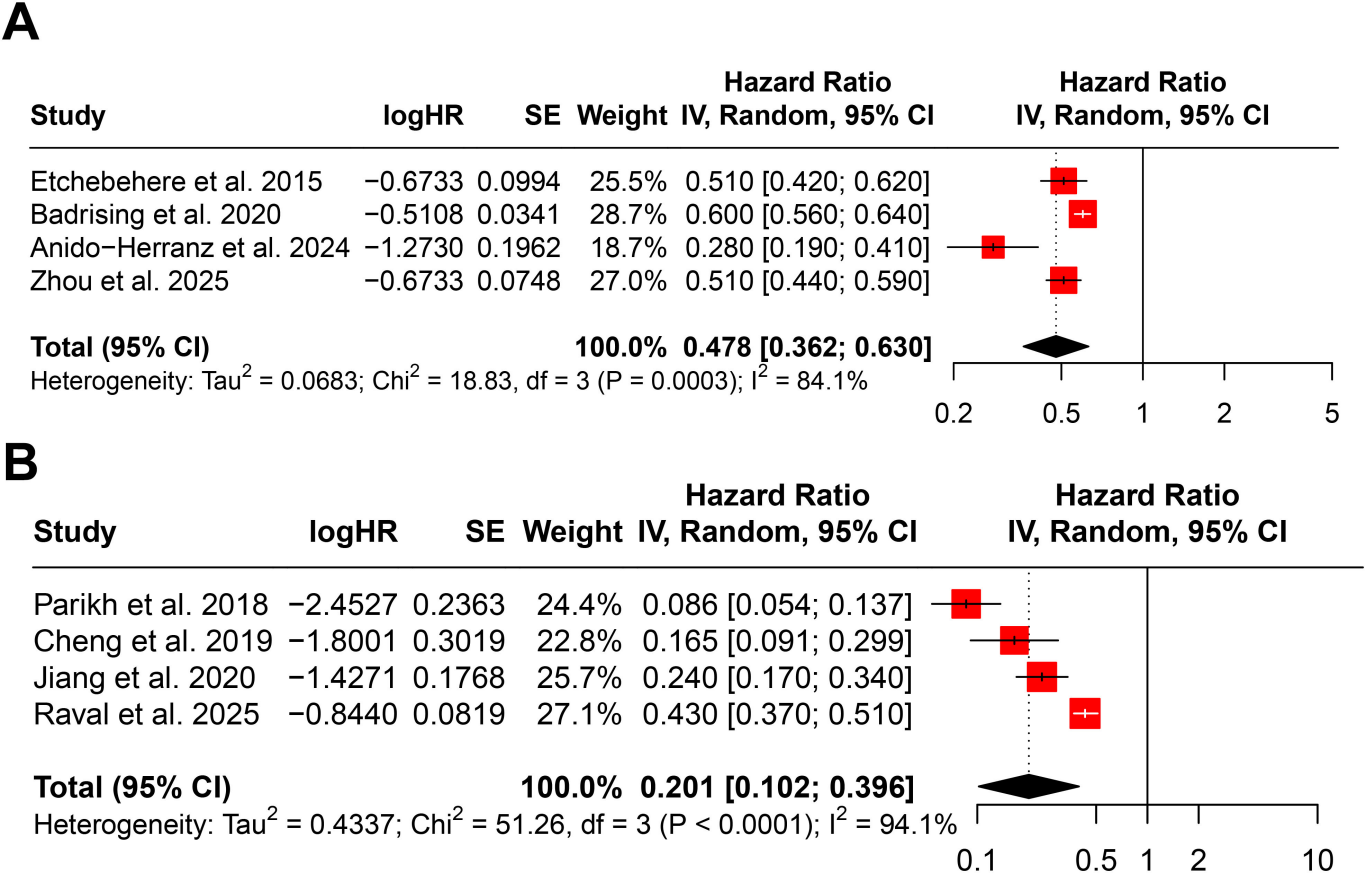
Forest plots showing the association between Ra-223 injection count and overall survival. **(A)** Ra-223 injection count as a continuous variable. **(B)** Ra-223 injection count as a binary variable (more *vs*. fewer injections).

The impact of various clinical and laboratory factors on OS following Ra-223 treatment was evaluated based on HRs extracted from the included studies. A total of 27 potential prognostic indicators were analyzed. The pooled results are summarized in **Table 2**, and representative forest plots are presented in **Figure 3** and **Supplementary Figures S2**-**S7**.

**Table 2:** Summary of pooled results.

Factor	Number of studies	Pooled HR	95% CI	P value	I²
Ra-223 Injection count (+1)	4	0.478	0.362-0.630	<0.001	84.1%
Ra-223 Injection count (More *vs*. Less)	4	0.201	0.102-0.396	<0.001	94.1%
Age (+1 year)	9	1.012	1.005-1.020	0.002	38.4%
Age (Older *vs*. Younger)	3	1.339	1.166-1.537	<0.001	0
ECOG (+1)	5	1.520	1.382-1.673	<0.001	0
ECOG (Higher *vs*. Lower)	6	2.078	1.791-2.425	<0.001	9.7%
PSA (Higher *vs*. Lower)	3	1.922	1.577-2.343	<0.001	0
PSA decline (Yes *vs*. No)	2	0.386	0.211-0.706	0.002	0
ALP (Higher *vs*. Lower)	7	1.981	1.708-2.298	<0.001	0
ALP decline (Yes *vs*. No)	2	0.701	0.504-0.975	0.035	0
LDH (Higher *vs*. Lower)	2	1.702	1.275-2.272	<0.001	0
logLDH (+1)	2	2.432	1.437-4.116	<0.001	78.3%
NLR (+1)	2	1.140	1.083-1.200	<0.001	0
NLR (Higher *vs*. Lower)	3	2.255	1.545-3.292	<0.001	62.6%
Hb (+1 g/dL)	8	0.756	0.699-0.816	<0.001	0.816
Hb (Higher *vs*. Lower)	6	0.456	0.367-0.566	<0.001	0
Neutrophil count (+1000/μL)	2	1.085	1.022-1.153	0.008	0
BSI (+1%)	2	1.428	0.910-2.240	0.121	97.7%
Gleason score (+1)	4	0.999	0.932-1.071	0.976	48.3%
Gleason score (Higher *vs*. Lower)	3	0.982	0.586-1.643	0.944	70.5%
Visceral metastasis (Yes *vs*. No)	3	2.342	1.338-4.099	0.003	69.8%
Lymph node involvement (Yes *vs*. No)	3	1.207	1.021-1.426	0.027	0
Prior skeletal events (Yes *vs*. No)	3	1.240	1.102-1.396	<0.001	22.0%
Prior chemotherapy (Yes *vs*. No)	8	1.425	1.282-1.583	<0.001	36.0%
Prior radiotherapy (Yes *vs*. No)	2	1.147	0.745-1.766	0.533	0
Concurrent abiraterone use (Yes *vs*. No)	2	0.566	0.107-2.990	0.503	90.3%
Concurrent bone protectants (Yes *vs*. No)	4	1.023	0.798-1.311	0.858	69.8%

Ra-223, radium-223; ECOG, Eastern Cooperative Oncology Group performance status; PSA, prostate-specific antigen; ALP, alkaline phosphatase; LDH, lactate dehydrogenase; NLR, neutrophil-to-lymphocyte ratio; Hb, hemoglobin; BSI, bone scan index; HR, hazard ratio; CI, confidence interval.

#### 3.3.1 Ra-223 injection count

Higher numbers of Ra-223 injections were significantly associated with improved OS. When analyzed as a continuous variable, each additional injection was associated with a 52.2% reduction in the risk of death (HR = 0.478, 95% CI: 0.362–0.630; P < 0.001; I² = 84.1%) (**Figure 3A**). As a binary variable (more *vs*. fewer injections), the HR was 0.201 (95% CI: 0.102–0.396; P < 0.001; I² = 94.1%) (**Figure 3**).

#### 3.3.2 Demographics and performance status

Increasing age was associated with statistically significant worse prognosis (HR per +1 year = 1.012, 95% CI: 1.005–1.020; P = 0.002). When analyzed categorically, older patients had a 33.9% higher risk of death compared to younger ones (HR = 1.339, P < 0.001; I² = 0) (**Supplementary Figures S2A, B**). Poor performance status, measured by Eastern Cooperative Oncology Group (ECOG) score, was a strong predictor of worse OS. The pooled HR was 1.520 per point increase in ECOG score (95% CI: 1.382–1.673; P < 0.001), and 2.078 when comparing patients with higher *vs*. lower ECOG scores (**Supplementary Figures S2C, D**).

#### 3.3.3 Laboratory biomarkers

Prostate-specific antigen (PSA) and alkaline phosphatase (ALP) were two of the most frequently reported markers of tumor burden in the included studies. Higher baseline PSA was significantly associated with poorer OS, with a pooled HR of 1.922 (95% CI: 1.577–2.343; P < 0.001) (**Supplementary Figure S3A**). In contrast, a decline in PSA during treatment was strongly predictive of improved survival (HR = 0.386, 95% CI: 0.211–0.706; P = 0.002) (**Supplementary Figure S3B**). Similarly, higher baseline ALP levels were linked to worse prognosis (HR = 1.981, 95% CI: 1.708–2.298; P < 0.001) (**Supplementary Figure S3C**), while patients who experienced a decline in ALP during therapy had significantly better OS (HR = 0.701, 95% CI: 0.504–0.975; P = 0.035) (**Supplementary Figure S3D**). High levels of lactate dehydrogenase (LDH) also correlated with worse survival. The pooled HR was 1.702 (95% CI: 1.275–2.272; P < 0.001; **Supplementary Figure S4A**). When analyzed as a continuous variable using log-transformed LDH values, the HR was 2.432 per unit (95% CI: 1.437–4.116; P < 0.001; **Supplementary Figure S4B**).

Higher neutrophil-to-lymphocyte ratio (NLR) were predictive of worse outcomes (NLR + 1 unit: HR = 1.140; P < 0.001; NLR high *vs*. low: HR = 2.255; **Supplementary Figures S4C, D**). Hemoglobin level was a strong protective factor (per +1 g/dL: HR = 0.756; high *vs*. low: HR = 0.456; both P < 0.001) (**Supplementary Figures S5A, B**). Elevated neutrophil counts also predicted poorer OS (HR = 1.085 per +1000/μL; P = 0.008) (**Supplementary Figure S5C**).

#### 3.3.4 Clinicopathological features

The bone scan index (BSI), which quantifies skeletal tumor burden, demonstrated a non-significant trend toward worse OS with increasing burden. The pooled hazard ratio per 1% increase in BSI was 1.428 (95% CI: 0.910–2.240; P = 0.121; I² = 97.7%) (**Supplementary Figure S5D**), indicating substantial heterogeneity and lack of statistical significance.

The Gleason score was not significantly associated with OS. When analyzed as a continuous variable, the pooled HR per +1 point increase was 0.999 (95% CI: 0.932–1.071; P = 0.976; **Supplementary Figure S6A**). Similarly, when dichotomized (higher *vs*. lower score), the association remained non-significant (HR = 0.982, 95% CI: 0.586–1.643; P = 0.944; **Supplementary Figure S6B**).

Metastatic distribution showed significant prognostic impact. The presence of visceral metastases was associated with markedly poorer survival (HR = 2.342, 95% CI: 1.338–4.099; P = 0.003; **Supplementary Figure S6C**). Lymph node involvement was also a significant risk factor, albeit with a smaller effect size (HR = 1.207, 95% CI: 1.021–1.426; P = 0.027; **Supplementary Figure S6D**). Prior skeletal events was also a significant predictor of poorer outcomes, with affected patients showing a 24.0% increased mortality risk (HR = 1.240, 95% CI: 1.102-1.396; P < 0.001; **Supplementary Figure S6E**).

#### 3.3.5 Prior and concurrent therapies

Treatment history analysis revealed that patients with prior chemotherapy exposure had substantially worse survival (HR = 1.425, 95% CI: 1.282-1.583; P < 0.001; **Supplementary Figure S7A**), while prior radiotherapy showed no significant association (HR = 1.147, 95% CI: 0.745-1.766; P = 0.533; **Supplementary Figure S7B**). Concurrent therapies during Ra-223 treatment indicated that neither abiraterone use (HR = 0.566, 95% CI: 0.107-2.990; P = 0.503; **Supplementary Figure S7C**) nor bone protectants (HR = 1.023, 95% CI: 0.798-1.311; P = 0.858; **Supplementary Figure S7D**) showed significant survival benefits, though both exhibited considerable heterogeneity (I² = 90.3% and 69.8% respectively).

## 4 Discussion

This meta-analysis evaluated prognostic factors associated with OS in patients with mCRPC treated with Ra-223 by synthesizing real-world evidence. The results showed that higher Ra-223 injection counts, better performance status, favorable hematologic markers (e.g., hemoglobin levels), and declines in PSA or ALP during treatment were significantly associated with improved OS, while visceral metastases, prior chemotherapy, and elevated inflammatory markers (e.g., NLR, LDH) predicted poorer outcomes. Notably, Ra-223 completion rates varied substantially across regions, underscoring the importance of treatment adherence. This study provides evidence for identifying patients most likely to benefit from Ra-223.

The pooled completion rate for Ra-223 therapy is lower than those reported in clinical trials (35, 36), which underscores a critical gap between efficacy and real-world effectiveness. The notable variability in Ra-223 completion rates across real-world settings highlights treatment implementation challenges. This estimate should be interpreted with caution due to substantial heterogeneity among the included studies. Our analysis demonstrates that fewer than 60% of patients complete the full six-dose regimen, with particularly low adherence rates observed in certain healthcare systems like the United States (48%). This treatment attrition represents a significant lost opportunity, given our finding that each additional Ra-223 injection was independently associated with substantially improved survival outcomes. This high level of heterogeneity indicates that the true completion rate varies considerably between different patient cohorts and healthcare systems. Our subgroup analysis by country partially explains this variability. The geographic disparities in completion rates likely reflect differences in clinical monitoring practices, management of treatment-related toxicities, and healthcare system factors such as reimbursement policies and care coordination (37). The successful completion of Ra-223 therapy is contingent upon multiple interrelated factors that merit careful consideration. Foremost among clinical determinants are the hematologic toxicities, with anemia and thrombocytopenia emerging as predominant causes of premature treatment discontinuation. These hematologic complications frequently necessitate dose delays or permanent cessation, particularly when they coincide with pre-existing myelosuppression (38). These findings underscore the need for standardized protocols to monitor and manage treatment-related adverse events, particularly hematologic toxicities that frequently lead to premature discontinuation (8). Future quality improvement initiatives should focus on implementing closer monitoring during early treatment cycles, and developing predictive tools to identify patients at highest risk for non-completion.

Beyond treatment adherence, this meta-analysis reveals several modifiable biological and therapeutic factors that demonstrate prognostic significance for outcomes with Ra-223. Hemoglobin management emerges as a strong prognostic factor, with every 1 g/dL increase associated with a 24% mortality risk reduction. While this observation raises the hypothesis that anemia correction might enhance outcomes, it remains uncertain whether improving hemoglobin levels would specifically augment Ra-223 efficacy or simply reflect better overall health status. Randomized data are needed to confirm causality. The dynamic behavior of traditional biomarkers also presents prognostic importance: patients achieving PSA or ALP declines during therapy demonstrated striking survival benefits, implying that early on-treatment monitoring could serve as a pragmatic tool for response-adaptive strategies. The inflammatory milieu appears equally consequential, as evidenced by the 14% mortality increase per unit rise in NLR. This finding supports exploratory interventions targeting systemic inflammation with corticosteroids, COX-2 inhibition, or novel immunomodulators in selected high-risk patients (39). However, this mechanistic insight remains a hypothesis and necessitates testing in randomized trials. Oddly, while prior skeletal events portended worse prognosis, conventional bone-targeted protectants failed to show survival benefit, exposing a fundamental disconnect between prognostic markers and modifiable interventions in bone health management. The limited number of studies with available data might be a potential reason for this negative connection. Similarly, in randomized clinical trial setting, the bisphosphonate sodium clodronate did not significantly improve OS (40). As for the non-modifiable nature of factors like visceral metastases and prior chemotherapy exposure further highlight the imperative to optimize these adjustable parameters when selecting candidates for Ra-223. Moving forward, priority should be given to prospective validation of anemia correction protocols, biomarker-guided early switching algorithms, and combinatorial approaches addressing inflammation and bone metabolism—while remaining mindful that these associations, however compelling, currently represent prognostic rather than predictive relationships until interventional studies prove otherwise.

A key methodological aspect of this meta-analysis was the decision to pool exclusively unadjusted HRs for prognostic factors. This approach was chosen to enhance the generalizability of our findings across diverse real-world settings. In observational studies, the selection of variables for multivariable adjustment is highly heterogeneous, often impacted by data availability and local clinical practices. Combining estimates from inconsistently adjusted models could might compromise the validity of pooled results. By utilizing unadjusted estimates, we aimed to capture the raw association between each prognostic factor and overall survival, as it manifests in routine clinical practice and is influenced by varying analytical choices.

The real-world nature of this meta-analysis represents a significant strength, as it synthesizes data from diverse clinical settings beyond the controlled environment of randomized trials, thereby enhancing the generalizability of our findings. Real-world evidence captures the heterogeneity of patient populations, including those with comorbidities, varying disease burdens, and differing treatment histories, thus providing a more pragmatic assessment of Ra-223’s effectiveness in routine practice. This is particularly relevant for mCRPC, a disease with complex management needs and limited therapeutic options. However, the limitations inherent to real-world data must be acknowledged, including potential biases from unmeasured confounders and variability in data collection methods across studies. Despite our comprehensive search strategy and the absence of significant funnel plot asymmetry for the primary outcome, the potential for publication bias cannot be entirely ruled out, particularly for analyses involving fewer studies. Besides, the real-world nature of the included studies inherently involves variable data completeness and quality across different registries and cohorts, which may have led to incomplete adjustment for all relevant confounders. Lastly, the inclusion of both retrospective and prospective observational studies might introduce heterogeneity in patient selection, data collection methods, and follow-up protocols, which could affect the consistency and generalizability of the pooled estimates.

## 5 Conclusion

This meta-analysis of real-world evidence identifies key prognostic factors influencing overall survival in mCRPC patients treated with Ra-223, including treatment adherence, hematologic parameters, and dynamic biomarker responses. The findings underscore the importance of optimizing modifiable factors such as anemia management and early toxicity monitoring. These insights may aid in risk stratification, patient selection, and supportive care strategies to improve outcomes in this challenging disease setting.

## Data Availability

The original contributions presented in the study are included in the article/**Supplementary Material**. Further inquiries can be directed to the corresponding author.

## Supplementary material

Supplementary Figure 1 Additional analyses of Ra-223 completion rates. **(A)** Sensitivity analysis. **(B)** Subgroup analysis by country. **(C)** Cumulative meta-analysis over time. **(D)** Funnel plot for publication bias assessment.

Supplementary Figure 2 Forest plots evaluating the prognostic impact of demographics and performance status on overall survival. **(A)** Age analyzed as a continuous variable. **(B)** Age as a binary variable (older *vs*. younger patients). **(C)** ECOG performance status per +1 point. **(D)** ECOG performance status as a binary variable (higher *vs*. lower scores).

Supplementary Figure 3 Forest plots assessing the association of PSA and ALP with overall survival. **(A)** Baseline PSA levels (higher *vs*. lower). **(B)** PSA decline during treatment (yes *vs*. no). **(C)** Baseline ALP levels (higher *vs*. lower). **(D)** ALP decline during treatment (yes *vs*. no).

Supplementary Figure 4 Forest plots examining the prognostic value of LDH and NLR for overall survival. **(A)** Baseline LDH levels (higher *vs*. lower). **(B)** Log-transformed LDH as a continuous variable. **(C)** NLR as a continuous variable. **(D)** NLR as a binary variable (higher *vs*. lower).

Supplementary Figure 5 Forest plots showing the impact of hematologic markers and bone scan index (BSI) on OS. **(A)** Hemoglobin levels per +1 g/dL. **(B)** Hemoglobin levels as a binary variable (higher *vs*. lower). **(C)** Neutrophil count per +1000/μL. **(D)** BSI per +1% increase.

Supplementary Figure 6 Forest plots showing clinicopathological predictors of OS. **(A)** Gleason score per +1 point. **(B)** Gleason score as a binary variable (higher *vs*. lower). **(C)** Presence of visceral metastases (yes *vs*. no). **(D)** Lymph node involvement (yes *vs*. no). **(E)** History of prior skeletal events (yes *vs*. no).

Supplementary Figure 7 Forest plots showing the influence of prior and concurrent therapies on overall survival. **(A)** Prior chemotherapy exposure (yes *vs*. no). **(B)** Prior radiotherapy exposure (yes *vs*. no). **(C)** Concurrent abiraterone use (yes *vs*. no). **(D)** Concurrent bone protectant use (yes *vs*. no).

Supplementary Table 1 Summary of search strategy.
