# Supplementary material for: Prognostic factors for overall survival in patients with metastatic castration-resistant prostate cancer treated with radium-223: a meta-analysis of real-world evidence
Source: Front Oncol. 2025 Nov 19;15:1672802. doi: 10.3389/fonc.2025.1672802 (PMC12672256; doi:10.3389/fonc.2025.1672802)
Supplement: Supplementary Figure 1 — Additional analyses of Ra-223 completion rates. (A) Sensitivity analysis. (B) Subgroup analysis by country. (C) Cumulative meta-analysis over time. (D) Funnel plot for publication bias assessment. [file DataSheet1.docx]

**Supplementary Table S1. Summary of search strategy**

| Pubmed | ("prostatic neoplasms"[MeSH Terms] OR "prostatic neoplasm"[Title/Abstract] OR "prostate cancer"[Title/Abstract] OR "prostate cancers"[Title/Abstract] OR "prostate carcinoma"[Title/Abstract] OR "cancer of prostate"[Title/Abstract] OR "CRPC"[Title/Abstract] OR "castration-resistant prostate cancer"[Title/Abstract] OR "castration resistant prostate cancer"[Title/Abstract] OR "mCRPC"[Title/Abstract] OR "metastatic castration-resistant prostate cancer"[Title/Abstract])  AND  ("radium"[MeSH Terms] OR "radium"[Title/Abstract] OR "radium 223"[Title/Abstract] OR "radium-223"[Title/Abstract] OR "Ra-223"[Title/Abstract] OR "Ra 223"[Title/Abstract] OR "Ra223"[Title/Abstract] OR "Xofigo"[Title/Abstract]) |
| --- | --- |
| Web of Science | (TI=("prostate cancer" OR "prostate cancers" OR "prostate carcinoma" OR "prostatic neoplasm" OR "CRPC" OR "castration-resistant prostate cancer" OR "castration resistant prostate cancer" OR "mCRPC" OR "metastatic castration-resistant prostate cancer"))  AND  (TI=("radium" OR "radium 223" OR "radium-223" OR "Ra-223" OR "Ra 223" OR "Ra223" OR "Xofigo")) |
| Embase | ((("prostate cancer" OR "prostate cancers" OR "prostate carcinoma" OR "prostatic neoplasm" OR "CRPC" OR "castration-resistant prostate cancer" OR "castration resistant prostate cancer" OR "mCRPC" OR "metastatic castration-resistant prostate cancer"):ti))  AND  ((("radium" OR "radium 223" OR "radium-223" OR "Ra-223" OR "Ra 223" OR "Ra223" OR "Xofigo"):ti)) |
| Cochrane Library | ("prostate cancer":ti,ab,kw OR "prostate carcinoma":ti,ab,kw OR "prostatic neoplasm":ti,ab,kw OR "CRPC":ti,ab,kw OR "castration-resistant prostate cancer":ti,ab,kw OR "castration resistant prostate cancer":ti,ab,kw OR "mCRPC":ti,ab,kw)  AND  ("radium":ti,ab,kw OR "radium 223":ti,ab,kw OR "radium-223":ti,ab,kw OR "Ra-223":ti,ab,kw OR "Ra 223":ti,ab,kw OR "Ra223":ti,ab,kw OR "Xofigo":ti,ab,kw) |

**Supplementary Figures**


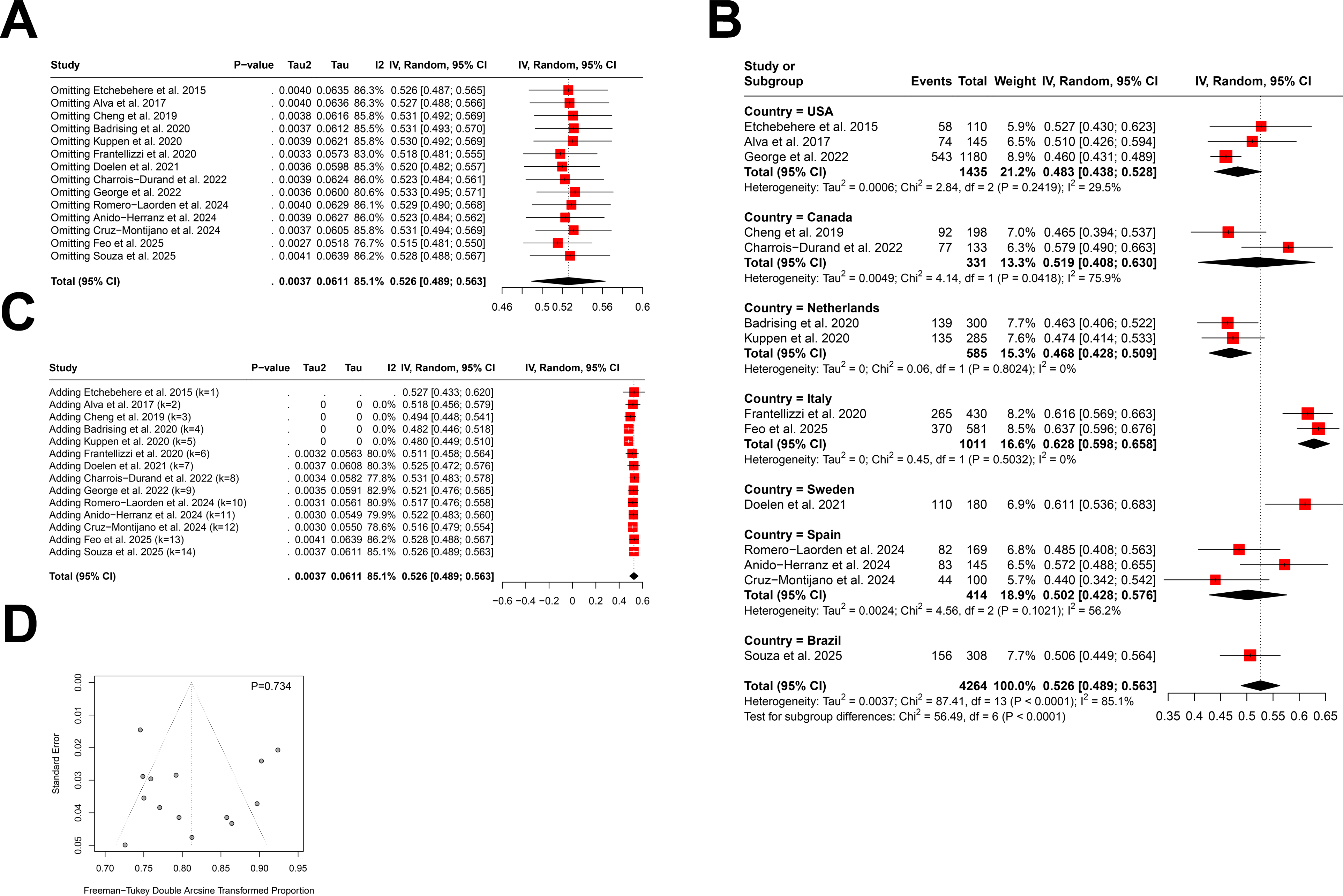


**Supplementary Figure S1. Additional analyses of Ra-223 completion rates.**

(A) Sensitivity analysis.

(B) Subgroup analysis by country.

(C) Cumulative meta-analysis over time.

(D) Funnel plot for publication bias assessment.


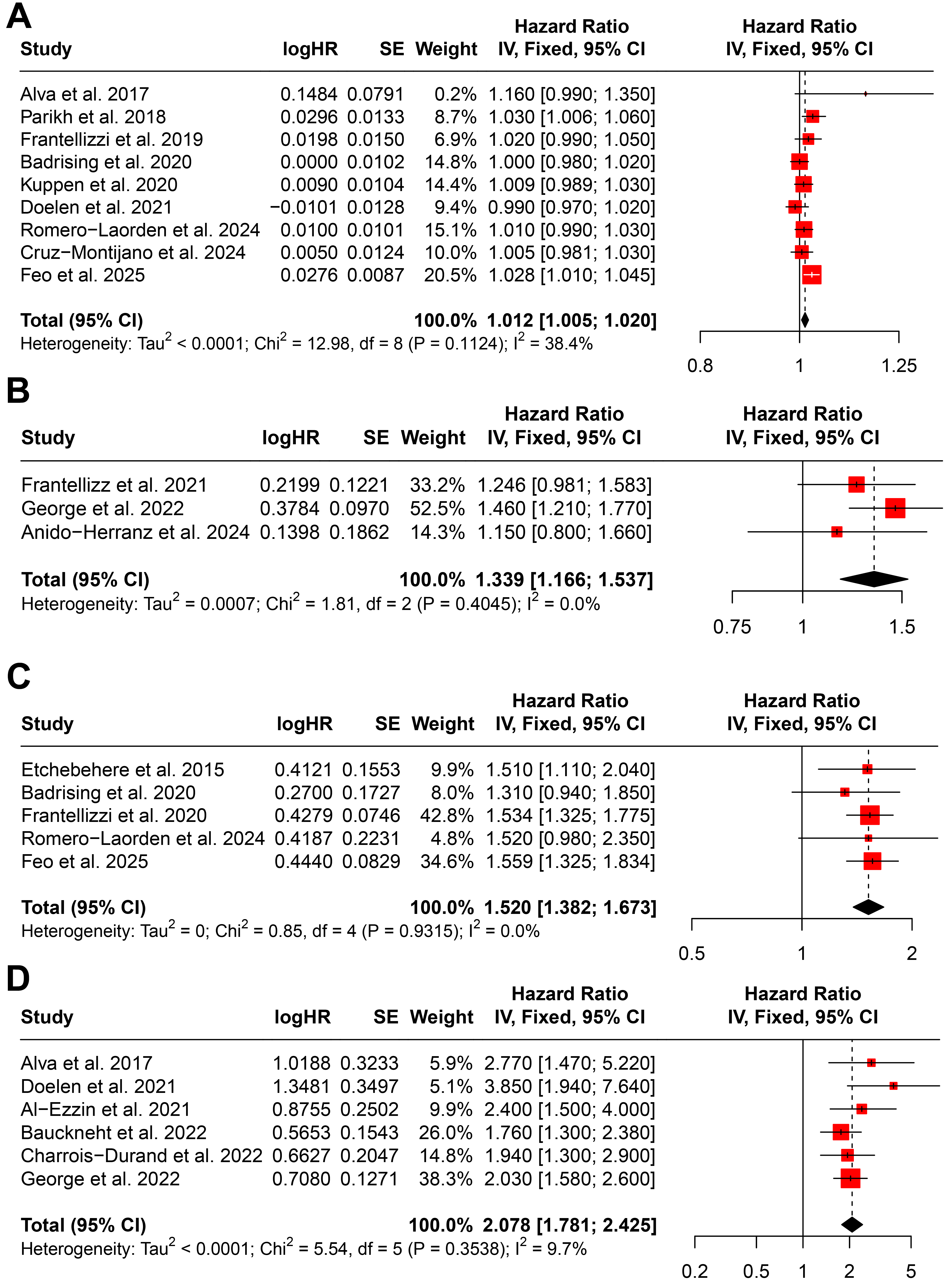


**Supplementary Figure S2. Forest plots evaluating the prognostic impact of demographics and performance status on overall survival.**

(A) Age analyzed as a continuous variable.

(B) Age as a binary variable (older vs. younger patients).

(C) ECOG performance status per +1 point.

(D) ECOG performance status as a binary variable (higher vs. lower scores).


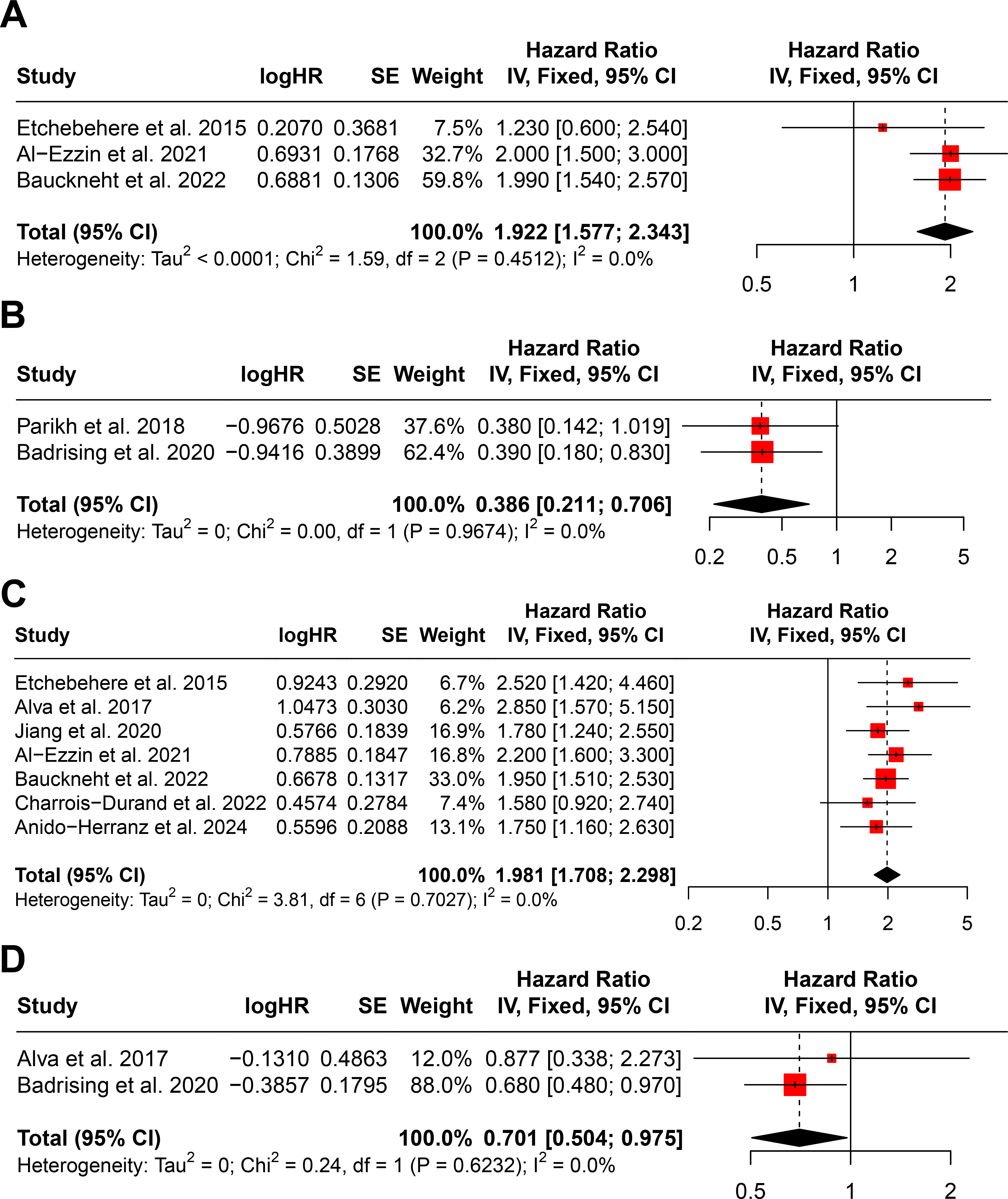


**Supplementary Figure S3. Forest plots assessing the association of PSA and ALP with overall survival.**

(A) Baseline PSA levels (higher vs. lower).

(B) PSA decline during treatment (yes vs. no).

(C) Baseline ALP levels (higher vs. lower).

(D) ALP decline during treatment (yes vs. no).


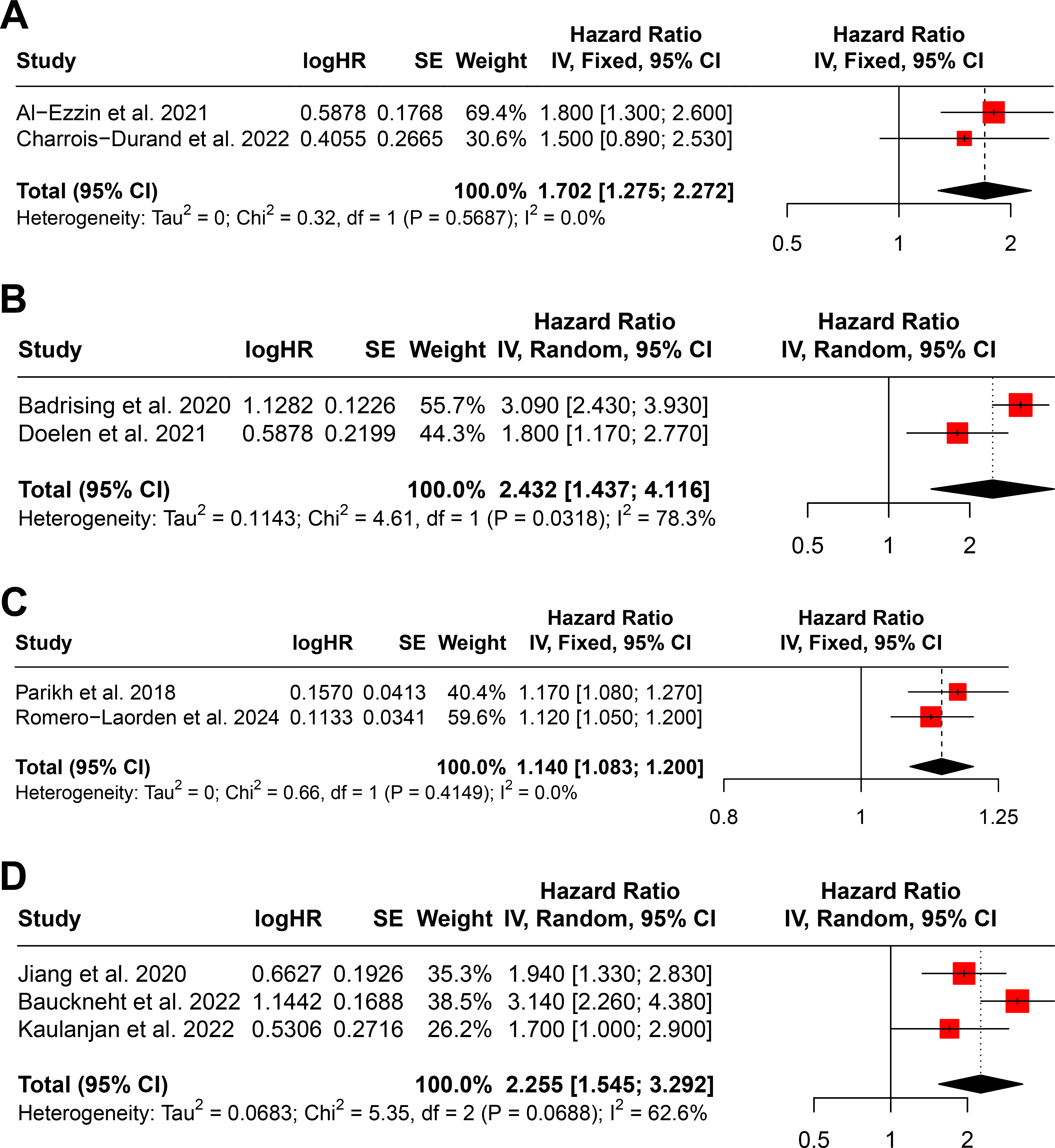


**Supplementary Figure S4. Forest plots examining the prognostic value of LDH and NLR for overall survival.**

(A) Baseline LDH levels (higher vs. lower).

(B) Log-transformed LDH as a continuous variable.

(C) NLR as a continuous variable.

(D) NLR as a binary variable (higher vs. lower).


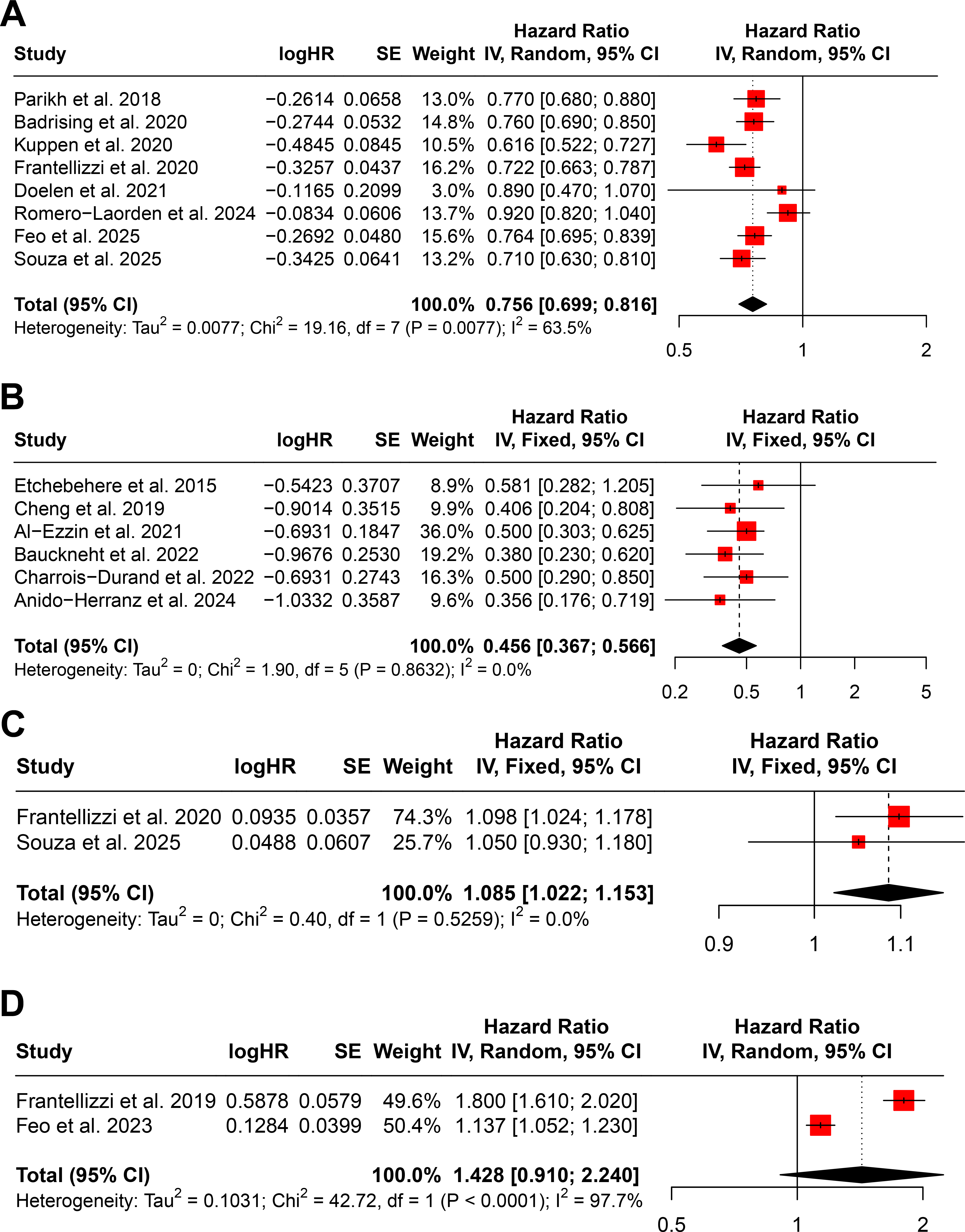


**Supplementary Figure S5. Forest plots showing the impact of hematologic markers and bone scan index (BSI) on OS.**

(A) Hemoglobin levels per +1 g/dL.

(B) Hemoglobin levels as a binary variable (higher vs. lower).

(C) Neutrophil count per +1000/μL.

(D) BSI per +1% increase.


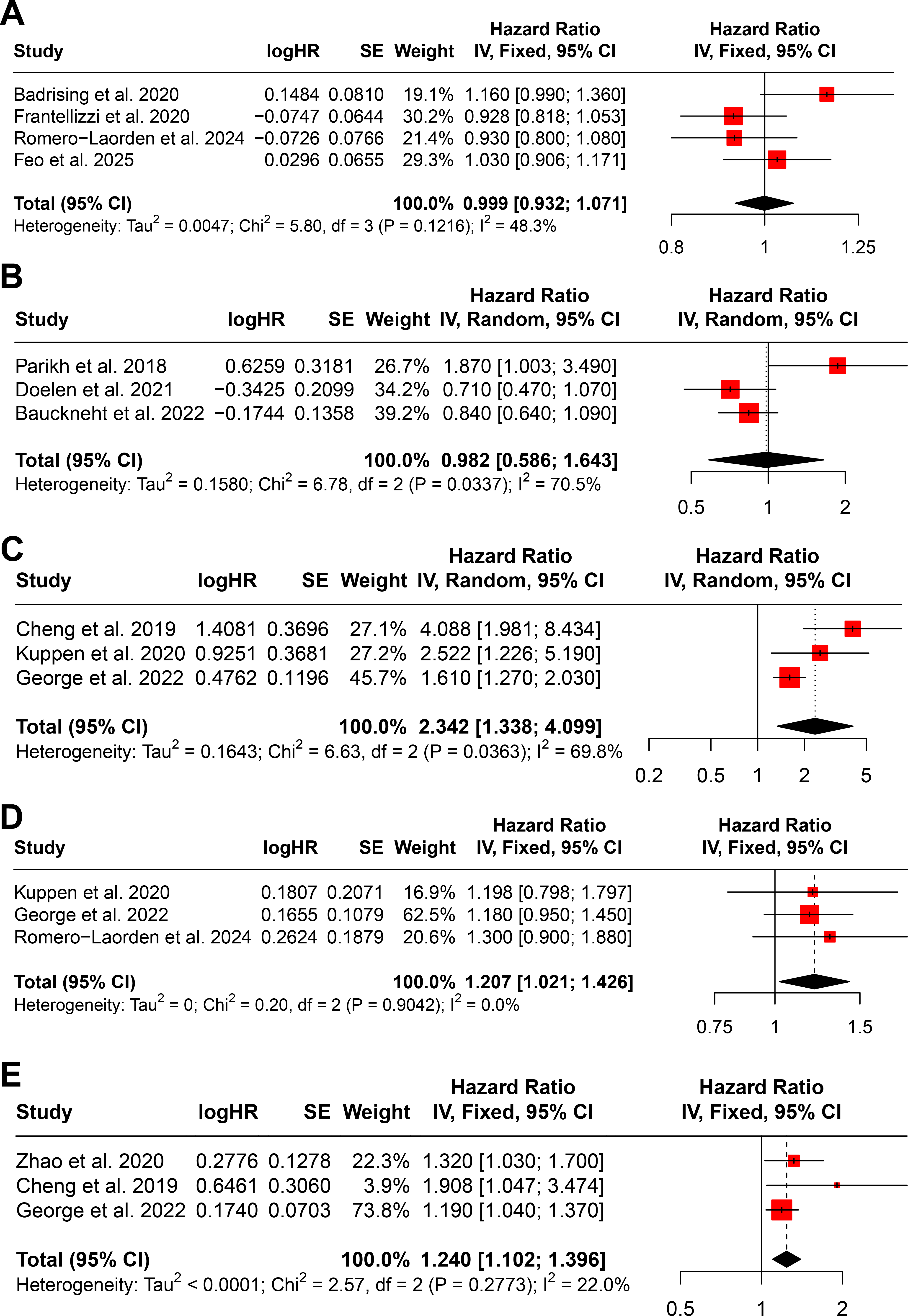


**Supplementary Figure S6. Forest plots showing clinicopathological predictors of OS.**

(A) Gleason score per +1 point.

(B) Gleason score as a binary variable (higher vs. lower).

(C) Presence of visceral metastases (yes vs. no).

(D) Lymph node involvement (yes vs. no).

(E) History of prior skeletal events (yes vs. no).


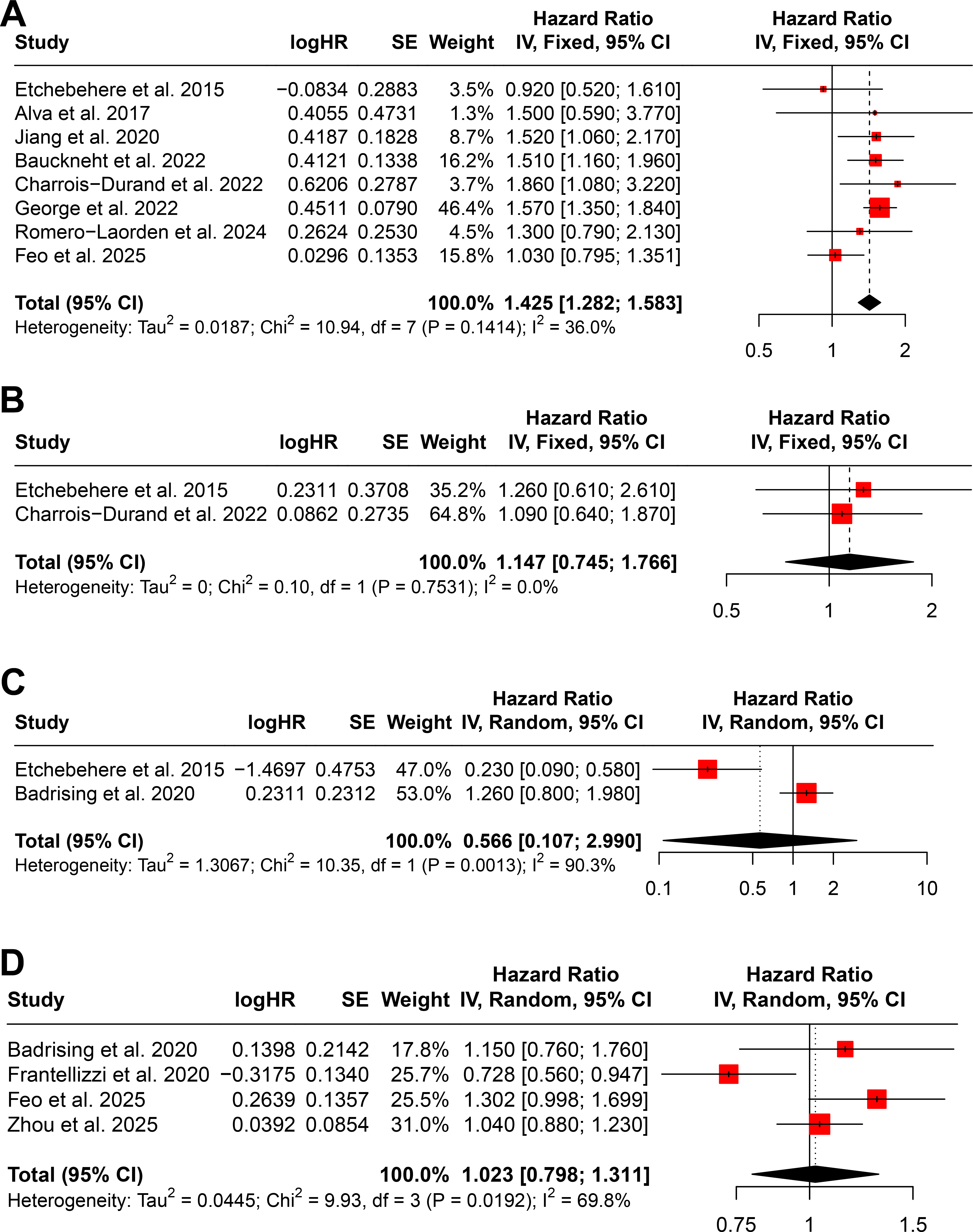


**Supplementary Figure S7. Forest plots showing the influence of prior and concurrent therapies on overall survival.**

(A) Prior chemotherapy exposure (yes vs. no).

(B) Prior radiotherapy exposure (yes vs. no).

(C) Concurrent abiraterone use (yes vs. no).

(D) Concurrent bone protectant use (yes vs. no).
